# 3D printed scaffolds of calcium silicate-doped β-TCP synergize with co-cultured endothelial and stromal cells to promote vascularization and bone formation

**DOI:** 10.1038/s41598-017-05196-1

**Published:** 2017-07-17

**Authors:** Yuan Deng, Chuan Jiang, Cuidi Li, Tao Li, Mingzheng Peng, Jinwu Wang, Kerong Dai

**Affiliations:** 1 Shanghai Key Laboratory of Orthopaedic Implants, Department of Orthopaedics, Shanghai Ninth People’s Hospital, Shanghai Jiao Tong University School of Medicine, Shanghai, 200011 China; 2 Med-X Research Institute, School of Biomedical Engineering, Shanghai Jiao Tong University, Shanghai, 200030 China; 3 Department of Orthopaedics, Sun Yat-sen Memorial Hospital, Sun Yat-sen University, Guangzhou, 510120 China

## Abstract

Synthetic bone scaffolds have potential application in repairing large bone defects, however, inefficient vascularization after implantation remains the major issue of graft failure. Herein, porous β-tricalcium phosphate (β-TCP) scaffolds with calcium silicate (CS) were 3D printed, and pre-seeded with co-cultured human umbilical cord vein endothelial cells (HUVECs) and human bone marrow stromal cells (hBMSCs) to construct tissue engineering scaffolds with accelerated vascularization and better bone formation. Results showed that *in vitro* β-TCP scaffolds doped with 5% CS (5%CS/β-TCP) were biocompatible, and stimulated angiogenesis and osteogenesis. The results also showed that 5%CS/β-TCP scaffolds not only stimulated co-cultured cells angiogenesis on Matrigel, but also stimulated co-cultured cells to form microcapillary-like structures on scaffolds, and promoted migration of BMSCs by stimulating co-cultured cells to secrete PDGF-BB and CXCL12 into the surrounding environment. Moreover, 5%CS/β-TCP scaffolds enhanced vascularization and osteoinduction in comparison with β-TCP, and synergized with co-cultured cells to further increase early vessel formation, which was accompanied by earlier and better ectopic bone formation when implanted subcutaneously in nude mice. Thus, our findings suggest that porous 5%CS/β-TCP scaffolds seeded with co-cultured cells provide new strategy for accelerating tissue engineering scaffolds vascularization and osteogenesis, and show potential as treatment for large bone defects.

## Introduction

Bone has high vascularity, for which early and efficient vascularization is important for the maintenance of cell survival, active remodeling and skeletal integrity^1^. Currently, pedicled grafts or bone supported by its own network of blood vessels are still the gold standard for treating large bone defects caused by trauma, tumour excision, and similar injuries. However, these are limited in availability, result in secondary damnification, and are often associated with donor site complications^2^. On the other hand, allografts and fabricated biomaterial scaffolds are associated with high failure rates of approximately 25%~60% in patients requiring large grafts, mainly due to slow vascularization and poor cellular viability (particularly at the core)^3, 4^. The emerging field of tissue engineering scaffolds with accelerated vascularization holds promise for the development of a much simpler solution for vascularized bone regeneration compared to the complicated pedicled and free vascularized allografts and synthetic grafts^4^.

Several strategies for improving the vascularization of synthetic scaffolds have been investigated with varying degrees of success. These can involve doped scaffolds with special elements^5, 6^. For example, silica and zinc oxide incorporated into three-dimensional (3D) printed β-tricalcium phosphate (β-TCP) scaffolds was shown to enhance angiogenesis *in vivo* ^7^. Other approaches have attempted to combine biomaterials with growth factors such as VEGF^8, 9^. These two kinds of approaches aim to produce a pro-angiogenic environment that can activate endogenous cells to vascularize the defect site and enhance repair. However, the host response might not occur in fast enough to allow grafts survival. Besides, vascular ingrowth is typically too slow because blood vessel invasion from the host is thought to occur at a rate of ~5 μm h^−1^.^10–12^ Another potential approach is to pre-seed scaffolds with specific cells to engineer a pro-angiogenic microenvironment *in vitro*, this would allow fast vascularization and integration after implantation. The formation of new blood vessels depends on the ordered interaction of endothelial cells (ECs) with perivascular cells and some other cells^13^. Human umbilical cord vein endothelial cells (HUVECs) have been shown to permit pre-vascularization of silk scaffolds and are highly effective at mobilizing to areas of ischemic damage and promoting neovascularization^14, 15^. Human bone marrow mesenchymal stem cells (HBMSCs) have been demonstrated to effectively stabilized nascent blood vessels *in vivo* by differentiating into perivascular cells^16^. Furthermore, hBMSCs could promote co-cultured HUVEC angiogenesis *in vitro* in comparison to single HUVECs through paracrine communication between HUVECs and hBMSCs^17^. Hence, in the study, co-cultured HUVECs and hBMSCs were selected as seed cells in an attempt to improve cellular viability and vascularization of synthetic scaffolds.

β-TCP, an ideal resorbable scaffold for bone regeneration, has already been used in the clinic^18^. Although β-TCP is biocompatible and osteoconductive, further improvements to angiogenesis and osteogenesis are needed^19–21^. In recent years, porous scaffolds containing calcium silicate (CS) have attracted much attention in the field of large bone repair to enhance vascularization, because silicon (Si) ions released from CS stimulate not only osteogenesis but also angiogenesis^22–25^. In light of the complementary characteristics of CS and β-TCP, the effective release of Si ions can promote angiogenesis *in vitro* by enhancing paracrine communication between co-cultured HUVECs and hBMSCs^17^. Porous β-TCP scaffolds doped with various ratios of CS were 3D printed and evaluated for biocompatibility and the ability to stimulate angiogenesis and osteogenesis.

In this study, to construct tissue engineering scaffolds with accelerated vascularization and functional integration into the host tissue after implantation, we investigated the indirect effects of composite scaffolds on angiogenesis in co-cultured HUVECs and hBMSCs cultured on Matrigel in real time. Subsequently, we pre-seeded co-cultured cells on scaffolds to study the direct relationship between scaffolds and co-cultured cells, including the spread morphology of co-cultured cells on scaffolds and whether optimized composite CS/β-TCP scaffolds could stimulate specific growth factors secretion into surrounding environment. Finally, we assessed vascularization and ectopic bone formation in the porous scaffolds seeded with co-cultured HUVECs and hBMSCs when transplanted subcutaneously into nude mice.

## Results

### Cell viability on 5%CS/β-TCP scaffolds

Composite scaffolds with different ratios of CS and β-TCP were fabricated with designed, well-controlled morphology and macroporous structure by 3D printing (Supplementary Fig. S1). Analysis of the phase composition by X-ray diffraction indicated that these composite scaffolds consisted of β-TCP and CS. Chemical reactions between components were not observed (Supplementary Fig. S2).

**Figure 1:**
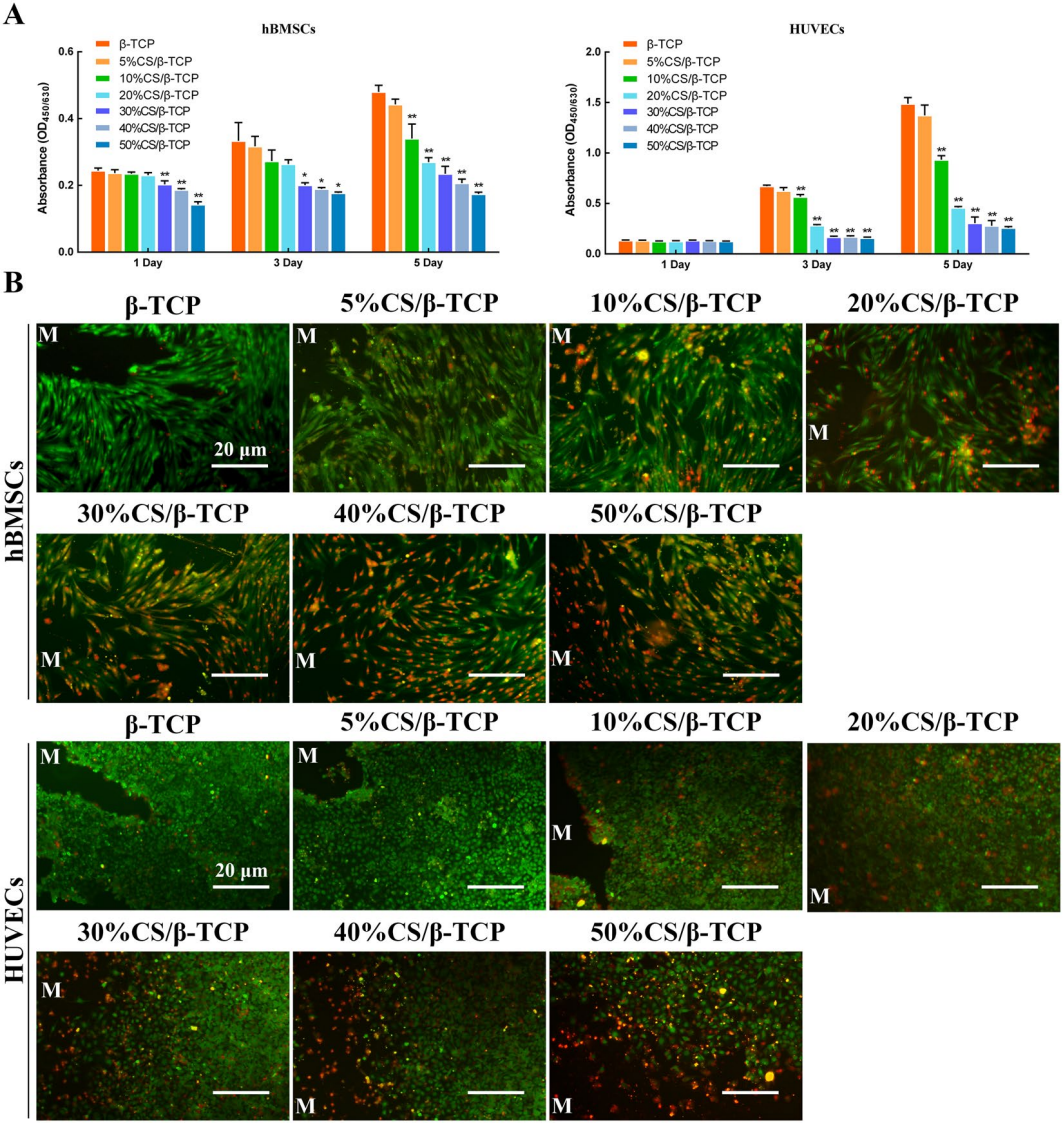
Cell viability on 5%CS/β-TCP scaffolds. (**A**) Cell viability, as measured by Cell Counting Kit-8, was comparable between β-TCP scaffolds and other composite scaffolds. Cell viability was significantly inhibited on scaffolds with more than 10% CS. (**B**) Cell viability of cells around scaffolds was also visualized by Live/Dead Kit, which stains viable cells green and dead cells red. *p < 0.05; **p < 0.01. Scale bar represents 20 μm. M marked materials.

Biocompatibility is a primary requirement for implanted biomaterials. Pure CS is poorly biocompatible^17, 22^. Thus, HUVECs and hBMSCs were seeded on various porous composite scaffolds, and cell viability was assessed using a Cell Counting Kit-8. As shown in Fig. 1A, cell viability was comparable at all time points between β-TCP scaffolds and various composite scaffolds. Cell viability was not significantly different between β-TCP scaffolds and 5%CS/β-TCP scaffolds. However, metabolic activity and cell proliferation were gradually and significantly inhibited (*p* < 0.01; vs. pure β-TCP) as the proportion of CS exceeded 10%. Accordingly, staining with Live/Dead Kit was used to examine the influence of different composite scaffolds on surrounding cell viability (Fig. 1B). It showed that, in comparison to HUVECs and hBMSCs growing around pure β-TCP scaffolds, tightly packed cells also grew around 5%CS/β-TCP scaffolds and these were mostly viable (green), with few dead cells (red) in the medium. In contrast, cells became increasingly sparse as CS exceeded 10%, with the number of dead cells increasing accordingly. Taken together, the data indicate that 5%CS/β-TCP scaffolds have better biocompatibility than other materials, and were thus subsequently characterized more intensively.

### Characteristics of 5%CS/β-TCP scaffolds

**Figure 2:**
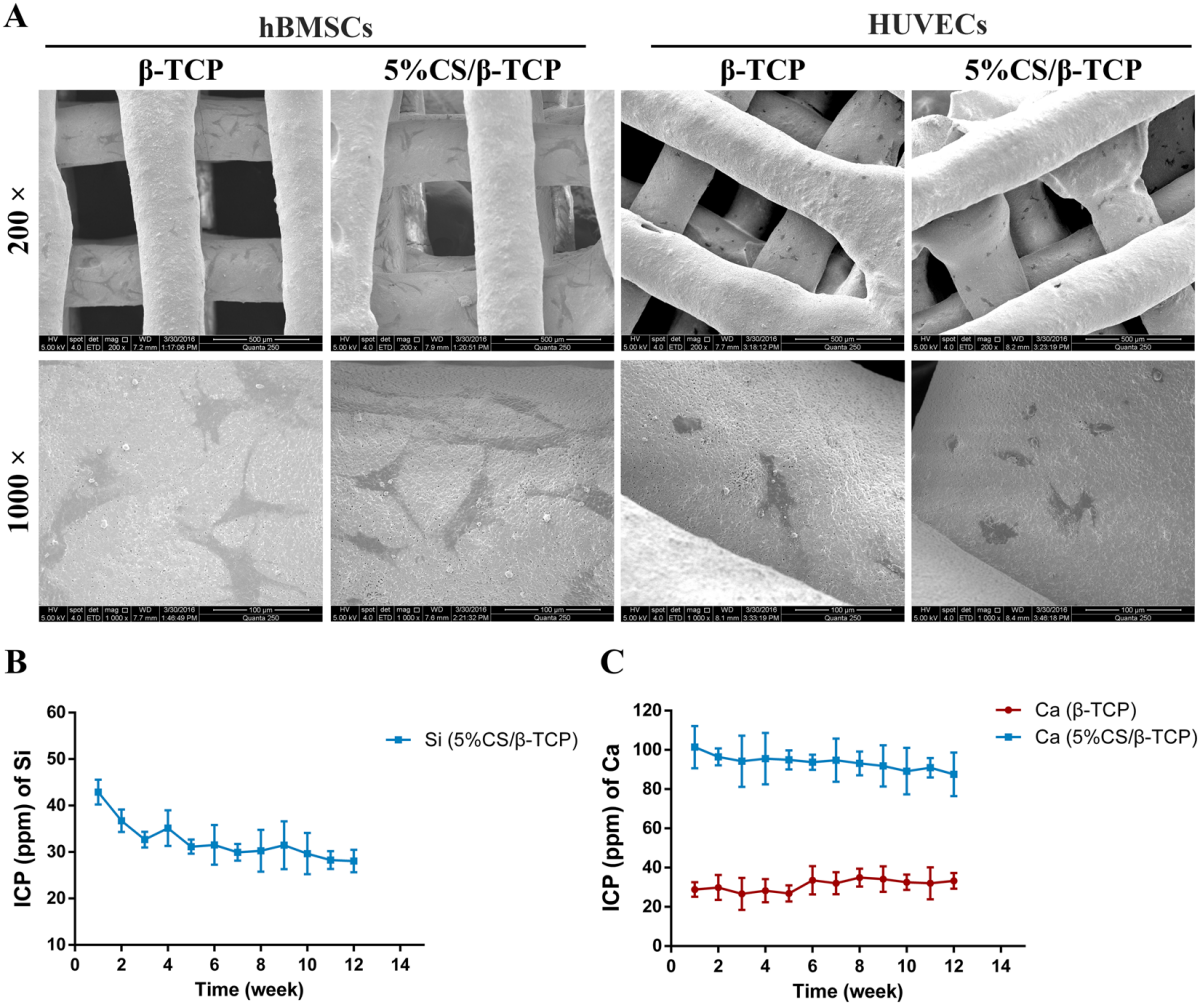
Characterization of 5%CS/β-TCP scaffolds. (**A**) SEM of HUVECs and hBMSCs seeded on 5%CS/β-TCP scaffolds. Release of (**B**) Si ions and (**C**) Ca ions. Scale bar represents 500 μm for 200﻿ X and 10﻿0 μ﻿m for 1000 X.

SEM indicated that the macropores in the scaffolds were approximately 350 μm, and that HUVECs and hBMSCs spread well on the surface of struts and inner pores of the 5%CS/β-TCP scaffold (Fig. 2A). Effective Si ion concentrations in the medium were shown to stimulate HUVEC angiogenesis. To study the release of ions when 5%CS/β-TCP scaffolds degraded (Supplementary Fig. S3), ICP was carried out. Results showed that release of Si ions was observed only from 5%CS/β-TCP scaffolds, and decreased with time (Fig. 2B). The amount of Ca ions released was much higher from 5%CS/β-TCP scaffolds (Fig. 2C).

### 5%CS/β-TCP scaffolds stimulate hBMSC osteogenesis and HUVEC angiogenesis *in vitro*

**Figure 3:**
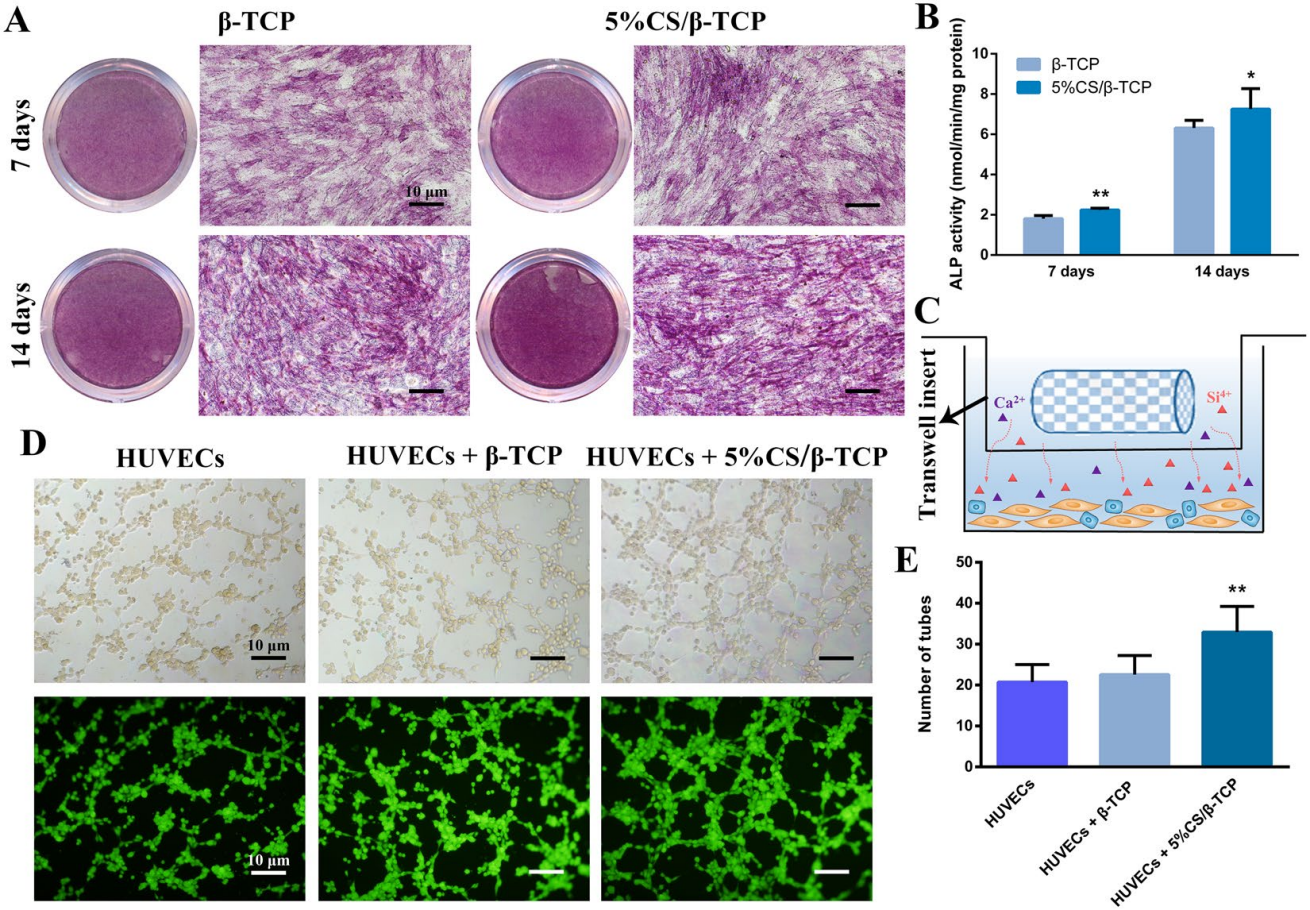
5%CS/β-TCP scaffolds stimulate hBMSC osteogenesis and HUVEC angiogenesis *in vitro*. (**A**) ALP staining in hBMSCs cultured for 7 and 14 days with β-TCP scaffolds or 5%CS/β-TCP scaffolds in transwell inserts. (**B**) 5%CS/β-TCP scaffolds stimulated ALP activity at 7 days and 14 days in comparison to pure β-TCP. (**C**) Schematic representation of transwell experiments. Scaffolds in Transwell inserts (upper chamber), cells in the lower chamber. (**D**) Tube formation by HUVECs, as observed by light microscopy (top) and Calcein AM staining (bottom), after 4 h on Matrigel with β-TCP or 5%CS/β-TCP scaffolds in transwell inserts. (**E**) 5%CS/β-TCP scaffolds enhanced tube formation in comparison to β-TCP scaffolds. *p < 0.05; **p < 0.01. Scale bar represents 10 μm.

To investigate the indirect effect of 5%CS/β-TCP scaffolds on hBMSCs in real time, scaffolds were seeded in transwell inserts and cells were seeded in the lower chamber (Fig. 3C). As shown in Fig. 3A, ALP staining showed that cells cultured with 5%CS/β-TCP in transwell inserts were intensively stained at 7 and 14 days. However, ALP staining was weakened with β-TCP in transwell inserts. Similarly, ALP activity at 7 and 14 days was higher in the presence of 5%CS/β-TCP than in the presence of pure β-TCP (Fig. 3B). Tube formation is a key step for angiogenesis *in vitro* ^26, 27^. To investigate the angiogenic effect of 5%CS/β-TCP scaffolds on HUVECs, cells were seeded on Matrigel in the lower chamber with 5%CS/β-TCP or β-TCP in transwell inserts. As seen in Fig. 3D and E, 5%CS/β-TCP scaffolds significantly stimulated tube formation in HUVECs cultured for 4 h on growth factor-reduced Matrigel, in comparison to that observed with pure β-TCP (*p < *0.01). Collectively, these results show that 5%CS/β-TCP scaffolds stimulate hBMSC osteogenesis and HUVEC angiogenesis in real time *in vitro*.

### 5%CS/β-TCP scaffolds enhance angiogenesis of co-cultured HUVECs and hBMSCs *in vitro*

**Figure 4:**
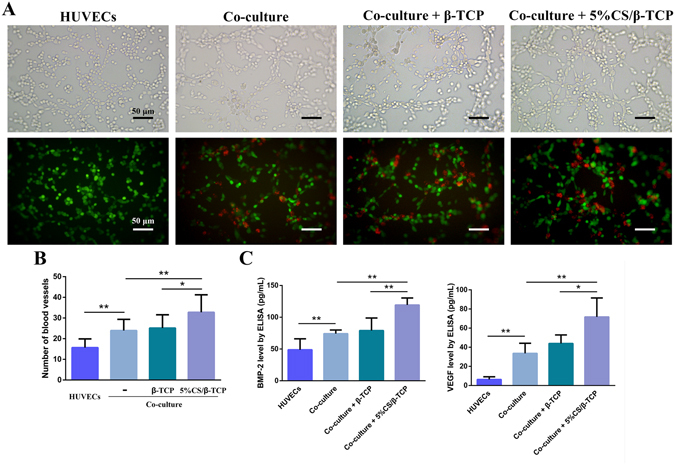
5%CS/β-TCP scaffolds enhanced angiogenesis in co-cultured HUVECs and hBMSCs. (**A**) Light microscopy (top) of tube formation on Matrigel, and fluorescence microscopy (bottom) of GFP-labelled HUVECs (green) stimulated for 4 h with β-TCP scaffolds or 5%CS/β-TCP scaffolds in transwell inserts, in the presence or absence of hBMSCs labelled with CM-Dil (red). (**B**) Co-culture of HUVECs with hBMSCs stimulated tube formation relative to monocultured HUVECs. 5%CS/β-TCP scaffolds further enhanced tube formation in co-cultured cells in comparison to pure β-TCP. (**C**) BMP-2 and VEGF secretion, as measured by ELISA. *p < 0.05; **p < 0.01. Scale bar represents 50 μm.

To track co-cultured cells, HUVECs were labelled with GFP, and hBMSCs were labelled with CM-Dil. As shown in Fig. 4A, tube formation was significantly enhanced when HUVECs (green) co-cultured with hBMSCs (red), as compared to that observed with HUVEC monocultures (*p* < 0.01). Tube formation was further stimulated (*p* < 0.01) by 5%CS/β-TCP scaffolds in transwell inserts, but not by pure β-TCP scaffolds (Fig. 4B). In addition, hBMSCs were clearly involved in tube formation, and were distributed evenly among HUVECs. In line with these results, ELISAs showed that VEGF and BMP-2 were more abundantly secreted (*p* < 0.01) in co-cultures than in monocultures, and that secretion was further enhanced by 5%CS/β-TCP scaffolds, but not by pure β-TCP (Fig. 4C).

### Formation of microcapillary-like structures

**Figure 5:**
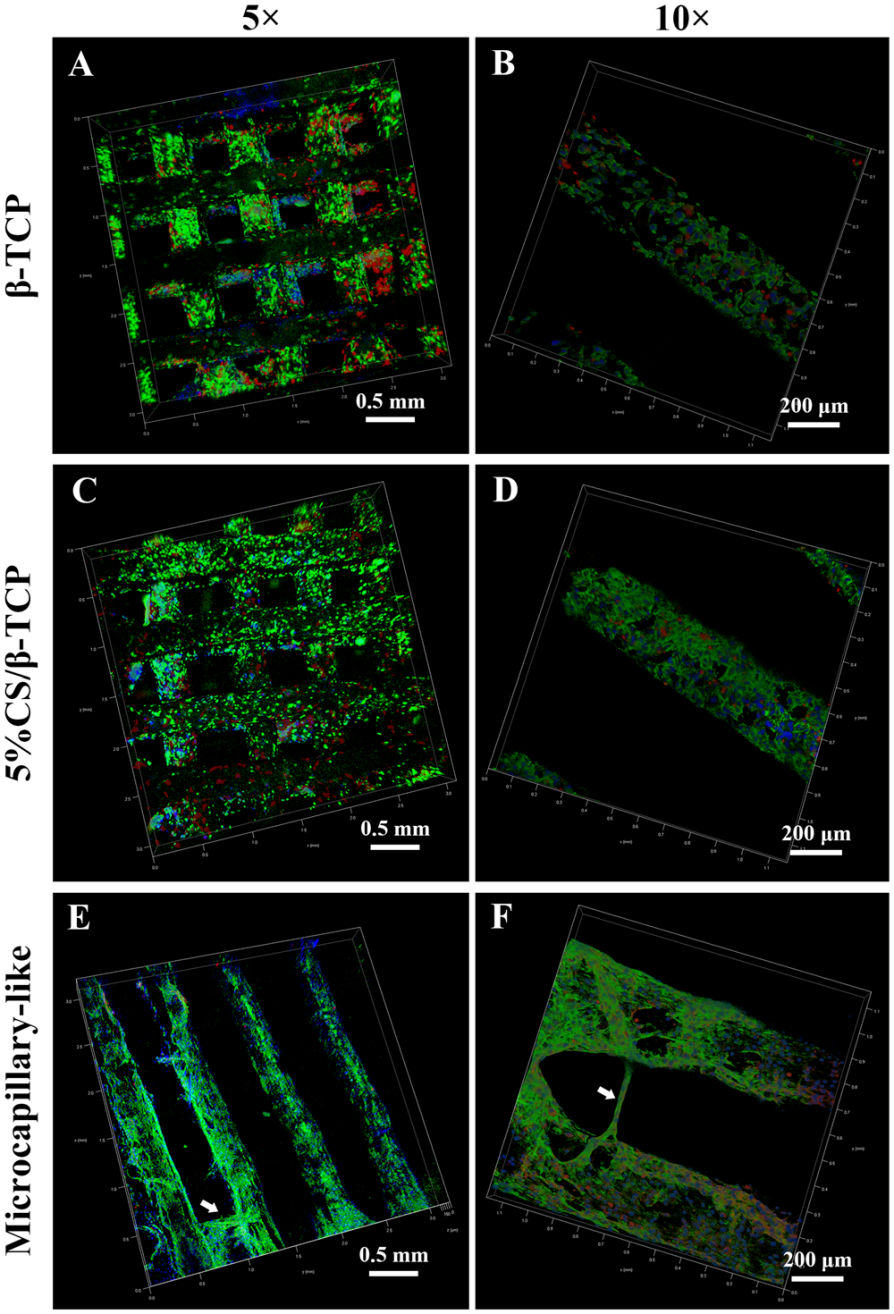
Confocal laser scanning microscopy of co-cultured cells on β-TCP and 5%CS/β-TCP scaffolds. Confocal images of HUVECs co-cultured with hBMSCs on (**A**,**B**) β-TCP and (**C**,**D**) 5%CS/β-TCP scaffolds for 3 days. Viable cells were stained with FITC (green), nuclei were stained with DAPI (blue), and hBMSCs were tracked with CM-Dil (red). (**E**,**F**) Confocal images of HUVECs co-cultured with hBMSCs labeled with CM-Dil (red) on 5%CS/β-TCP scaffolds for 10 days. Endothelial cells were stained with CD31 (green), and nuclei were stained with DAPI (blue). White arrows mark CD31-positive microcapillary-like structures.

As we noted, tube formation of co-cultured cells was further stimulated by 5%CS/β-TCP scaffolds; however, cells were physically separated by transwell inserts. Thus, we co-cultured HUVECs and hBMSCs directly on scaffolds to investigate spread morphology and cell interactions on 3D materials. Results showed that co-cultured cells proliferated well on β-TCP and 5%CS/β-TCP scaffolds, and spread well on the surface of struts and inner pores of scaffolds at three days (Fig. 5A–D). At 10 × magnification, hBMSCs labelled with CM-Dil (red) were observed to be evenly distributed among HUVECs (Fig. 5B,D). Immunofluorescence microscopy probing for CD31, which is expressed at the cell-cell interface, indicated that 5%CS/β-TCP scaffolds stimulated co-cultured cells to form microcapillary-like structures after 10 days (Fig. 5E,F). But microcapillary-like structures were not observed on β-TCP scaffolds at the same time. Besides, at 10 × magnification, these structures could be clearly observed, as judged by the location of nuclei, and by staining for HUVECs (green) and hBMSCs (red, Fig. 5F).

### 5%CS/β-TCP scaffolds enhance the migration of BMSCs toward co-cultured cells

**Figure 6:**
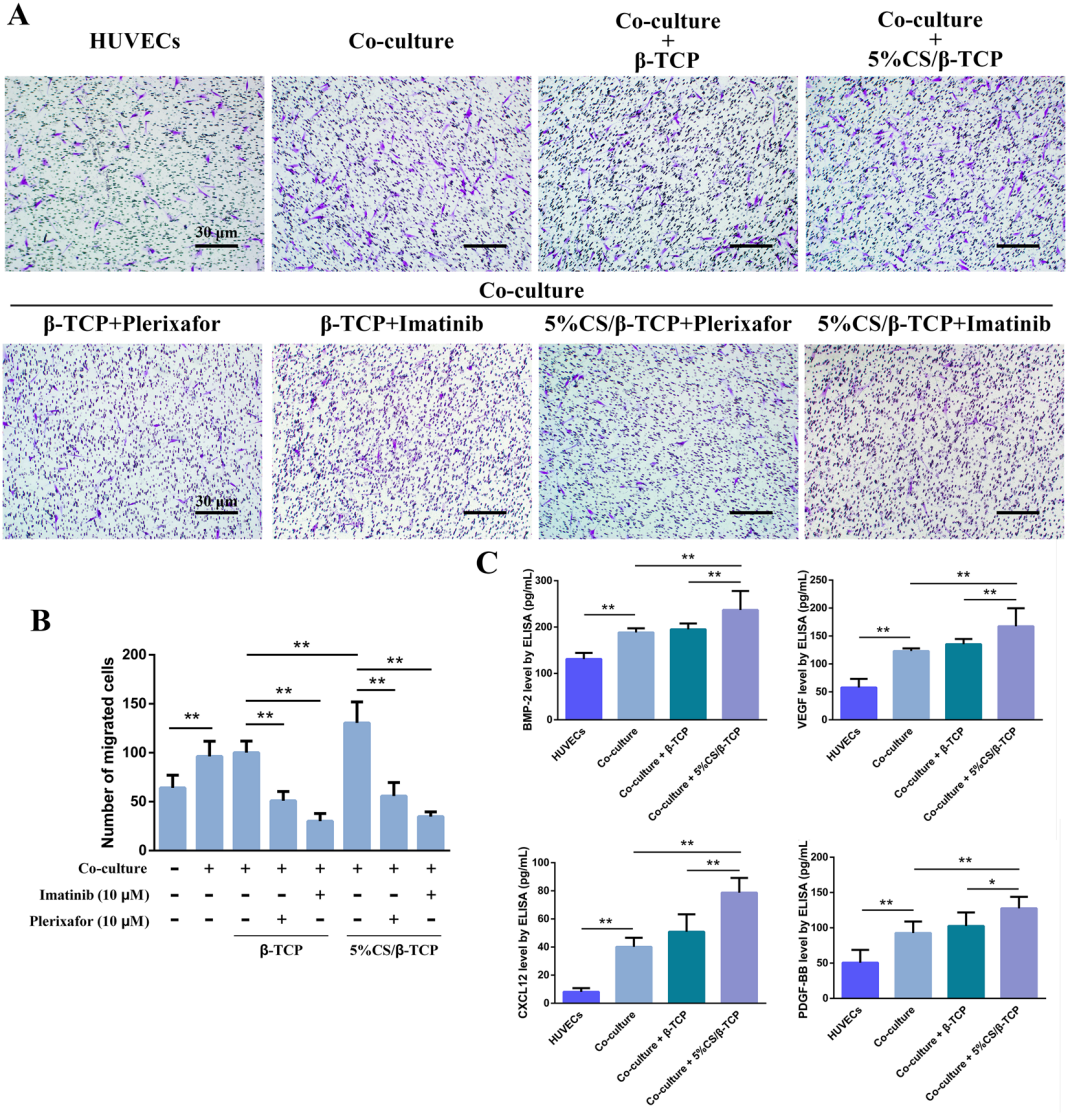
5%CS/β-TCP scaffolds enhance migration of hBMSCs. (**A**) Transwell migration assay with β-TCP scaffolds or 5%CS/β-TCP scaffolds, and with 10 μM of the inhibitors plerixafor or imatinib. (**B**) 5%CS/β-TCP scaffolds enhanced the migration of hBMSCs, an effect blocked by imatinib and plerixafor. (**C**) BMP-2, VEGF, CXCL12 and PDGF-BB secretion, as measured by ELISA. *p < 0.05; **p < 0.01. Scale bar represents 30 μm.

Angiogenesis is a complex process involving many factors and cells^28^. Our previous results showed that 5%CS/β-TCP scaffolds stimulated co-cultured cells to form microcapillary-like structures on the scaffolds. And studies have shown that HUVECs could recruit MSCs^16^. Hence, we tested whether 5%CS/β-TCP scaffolds stimulated co-cultured cells in direct contact to secrete some growth factors that enhance angiogenesis and migration of MSCs. hBMSCs were seeded in transwell inserts physically separated from 5%CS/β-TCP scaffolds seeded with co-cultured cells in the lower chamber, and ELISA was carried out. Results showed that 5%CS/β-TCP scaffolds stimulated co-cultured cells to secrete significant levels of VEGF and BMP-2 into the surrounding environment (Fig. 6C). Besides, as shown in Fig. 6A, co-culture of HUVECs and hBMSCs enhanced migration of hBMSCs from transwell inserts. Migration was further stimulated by 5%CS/β-TCP scaffolds, but not by pure β-TCP scaffolds (Fig. 6B). Similar results were obtained when mouse BMSCs were seeded in transwell inserts (data not shown). According to previous reports, factors like PDGF-BB and CXCL12 promote MSC migration^29, 30^. Indeed, imatinib, an inhibitor of PDGF-BB, significantly inhibited hBMSC migration, as did plerixafor, an inhibitor of CXCL12 (Fig. 6A and B). Further, ELISA confirmed that PDGF-BB and CXCL12 were abundantly secreted in co-cultures and that 5%CS/β-TCP scaffolds enhanced secretion (Fig. 6C).

### Rapid vascularization and new tissue formation *in vivo*

**Figure 7:**
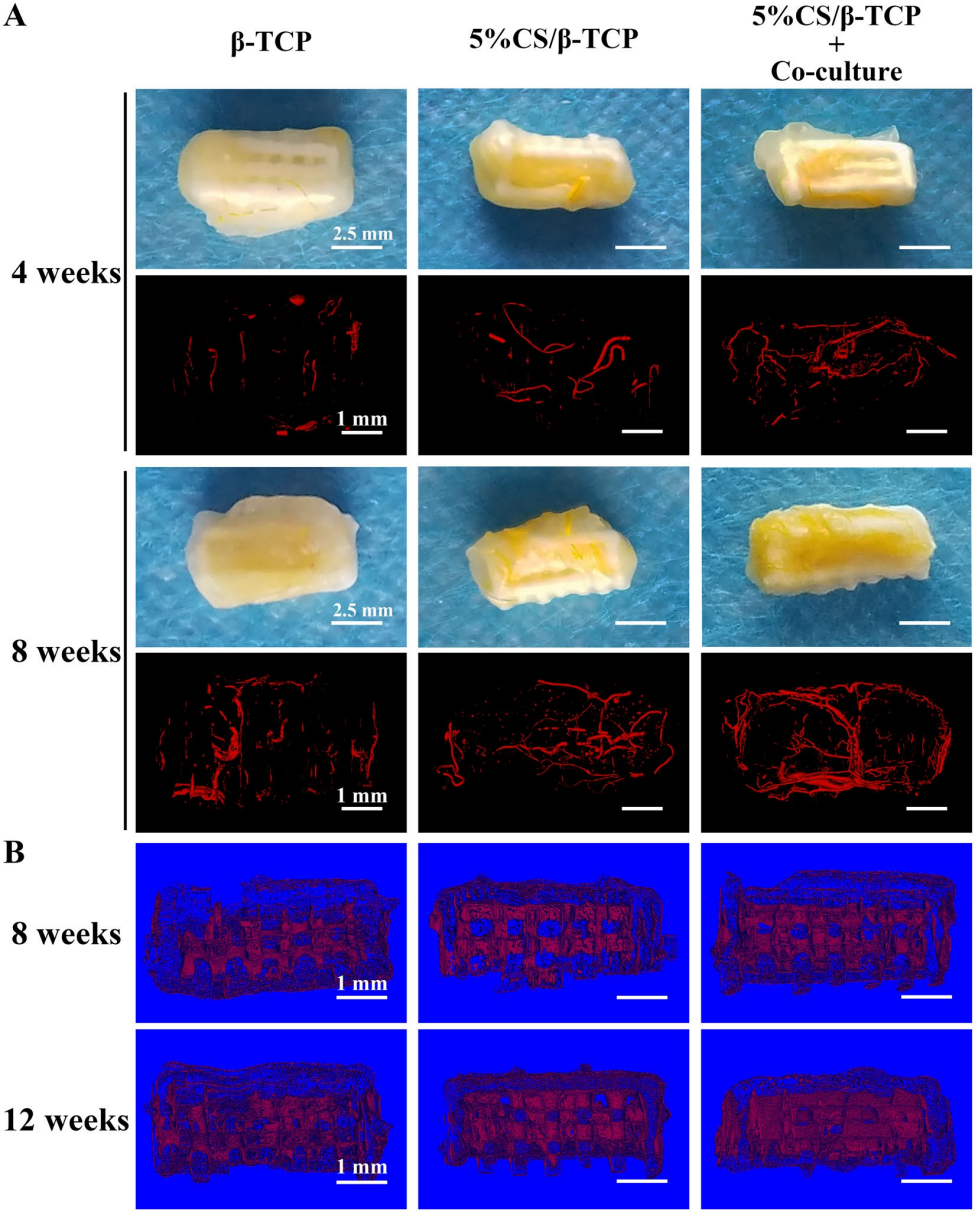
Analysis of new vessels and new tissue. (**A**) Macroscopic and micro-CT imaging of new vessels formed at 4 and 8 weeks in β-TCP and 5%CS/β-TCP scaffolds, as well as in 5%CS/β-TCP scaffolds seeded with co-culture cells. (**B**) Micro-CT analysis of new tissues formed in scaffolds at 8 and 12 weeks. Scale bar represents 2.5 mm for macroscopic and 1 mm for micro-CT.

New vessels were observed by micro-angiography and micro-CT at 4 and 8 weeks after subcutaneous implantation of 3D-printed scaffolds. Indeed, macroscopic (vessels were stained yellow by Microfil) and micro-CT 3D reconstructions (red) revealed direct vascularization of implants. More vessels were formed in 5%CS/β-TCP scaffolds than in pure β-TCP scaffolds. Moreover, pre-seeding with HUVECs and hBMSCs significantly enhanced and accelerated vascularization (Fig. 7A). New tissue was also observed by micro-CT at 8 and 12 weeks in all scaffolds, but this was most pronounced in the macropores of 5%CS/β-TCP scaffolds seeded with co-cultured cells (Fig. 7B).

### Vessel penetration and ectopic bone formation in macropores *in vivo*

**Figure 8:**
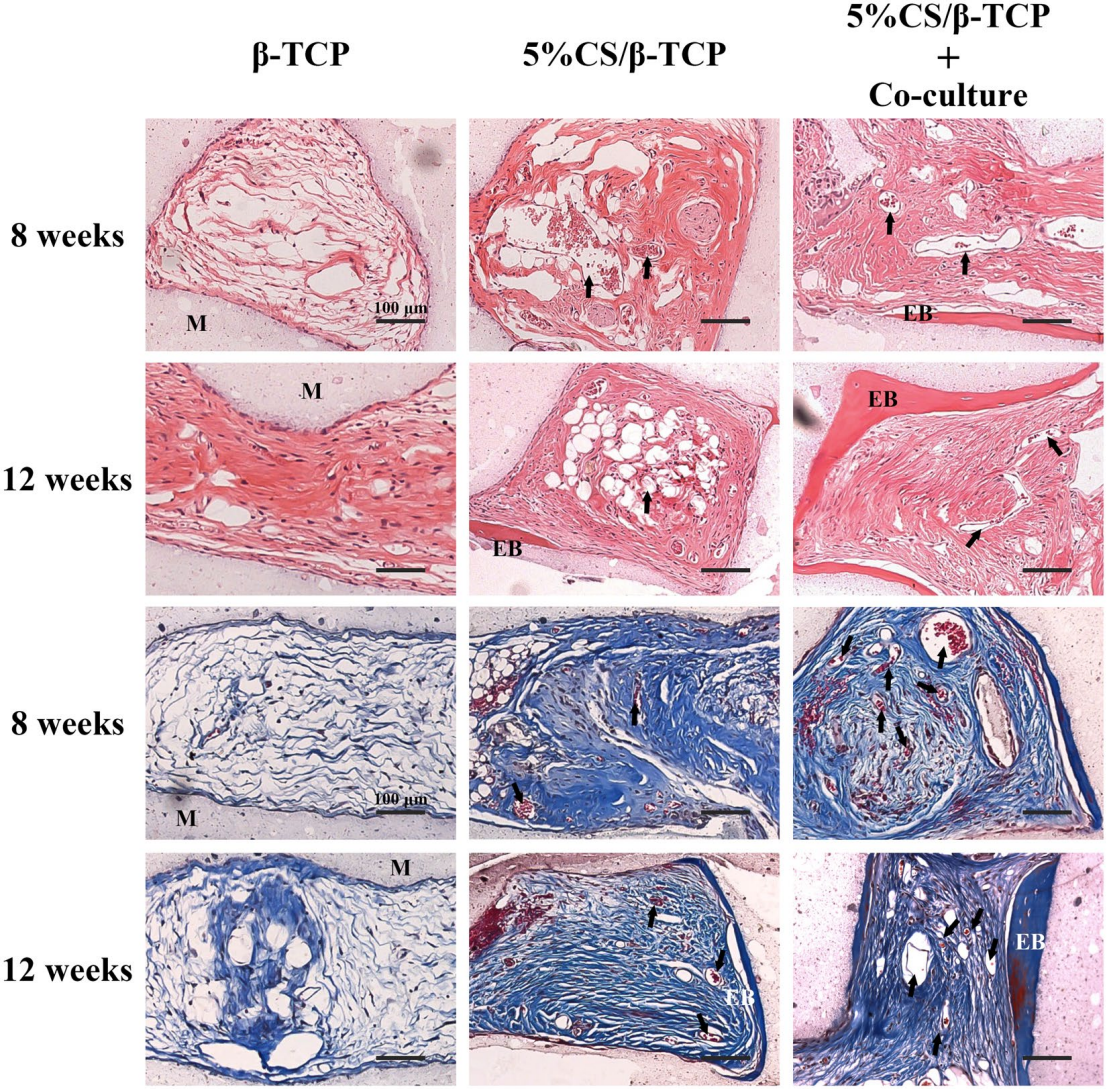
Histological observation of vessel penetration and ectopic bone formation on β-TCP and 5%CS/β-TCP scaffolds, as well as on 5%CS/β-TCP scaffolds pre-seeded with HUVECs and hBMSCs. Specimens were collected at 8 and 12 weeks, and stained with haematoxylin-eosin and Masson’s trichrome. Arrows indicate new vessels, materials is marked M, and ectopic bone is marked EB. Scale bar represents 100 μm.

Specimens were stained with haematoxylin-eosin and Masson’s trichrome to examine vessel and ectopic bone formation in the macropores of scaffolds at 8 and 12 weeks (Fig. 8). Few new blood vessels and loose fibrous tissue, but not bone tissue, were observed at both time points in the macropores of β-TCP scaffolds. In 5%CS/β-TCP scaffolds, connective tissue had formed and vascular penetration was observed at 8 weeks in the macropores, with some ectopic bone tissue formed at 12 weeks. However, many blood vessels and some ectopic bone had formed at 8 weeks in the macropores of 5%CS/β-TCP scaffolds seeded with co-cultured cells, with significant ectopic bone formation four weeks later. Besides, fewer cells were observed in the macropores of β-TCP scaffolds at 8 weeks. But much more cells were observed in the macrosopes of 5%CS/β-TCP scaffolds and 5%CS/β-TCP scaffolds pre-seeded co-cultured cells at 8 weeks. Collectively, the data indicated that doping β-TCP scaffolds with 5% CS accelerated host cell recruitment to the macropores of scaffolds, promoted vascularization and induced ectopic bone formation. Pre-seeding with HUVECs and hBMSCs further promoted vascularization and bone formation.

**Figure 9:**
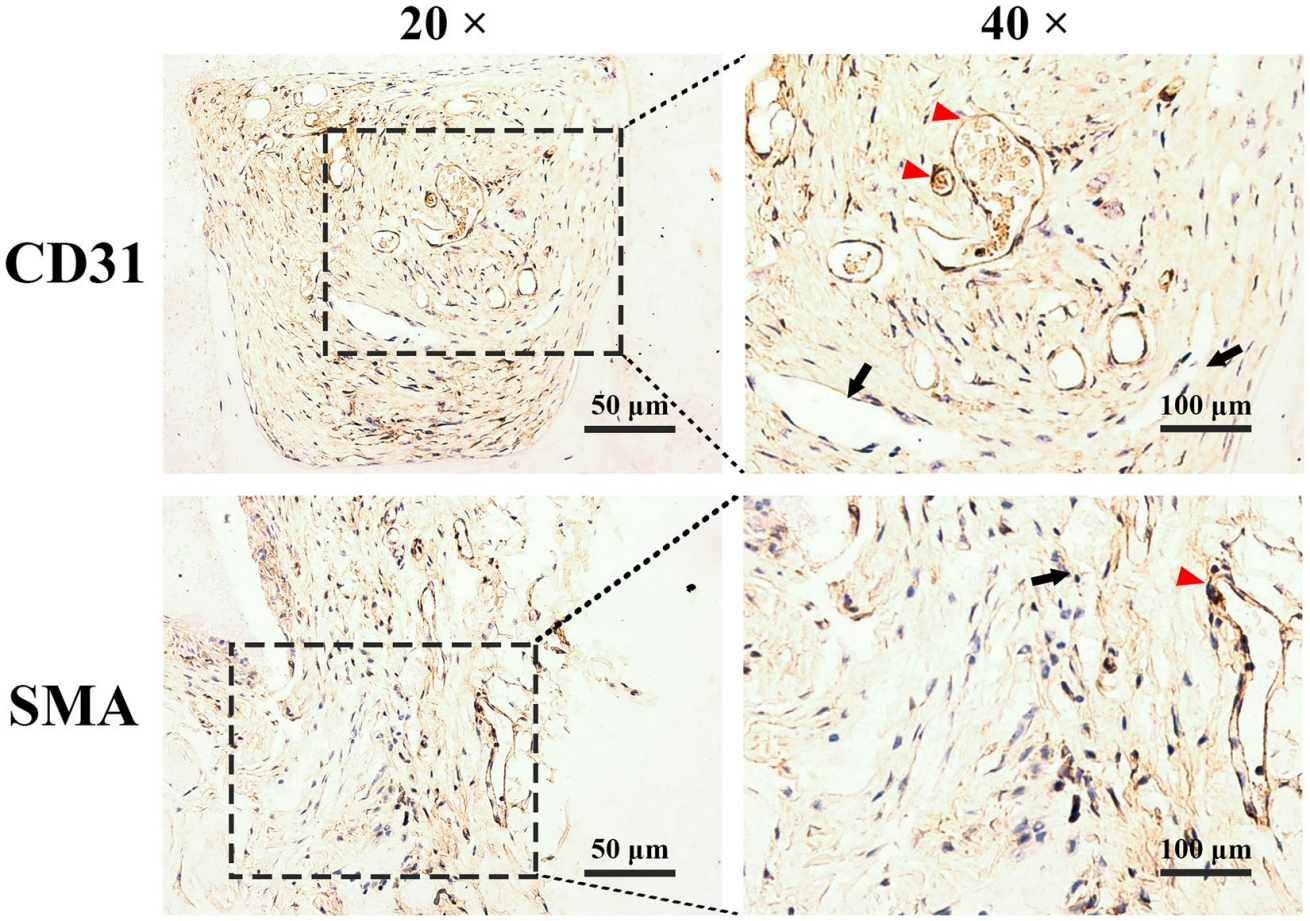
Representative immunohistological sections of 5%CS/β-TCP scaffolds pre-seeded with HUVECs and hBMSCs and implanted into nude mice. A few luminal structures contained human CD31 and human SMC (red arrows), but others did not (black arrows) and were solely host-derived.

Sections of implants were probed with antibodies against human CD31 and human SMC to trace pre-seeded HUVECs and hBMSCs, respectively. As shown in Fig. 9, a few luminal structures within the implant (red arrowheads) stained for human CD31, indicating that pre-seeded HUVECs were involved in the formation of these lumens. Pre-seeded hBMSCs were also detected around these lumens (red arrowheads), suggesting that exogenous hBMSCs also were involved in on-going vessel maturation. However, most luminal structures did not stain for CD31 and SMC (black arrows), indicating that most new vessels were invading murine blood vessels.

## Discussion

Insufficient vascularization remains a major challenge when using synthetic bone scaffolds to repair large bone defects^4, 13^. Therefore, this study sought to construct tissue engineering scaffolds with accelerated vascularization and functional integration into host tissue after implantation. Results showed that 3D-printed porous composite scaffolds that β-TCP scaffolds containing 5% CS were biocompatible, and stimulated angiogenesis and osteogenesis *in vitro* and *in vivo*. In addition, seeded co-cultured HUVECs and hBMSCs on 5%CS/β-TCP scaffolds, scaffolds stimulated co-cultured cells to secrete growth factors such as VEGF, BMP-2, PDGF-BB, CXCL12 into the surrounding environment. This indicated that a pro-angiogenic tissue engineering microenvironment was successfully constructed *in vitro*. *In vivo* assessment demonstrated that 5%CS/β-TCP scaffolds synergized with co-cultured HUVECs and hBMSCs to enhance early vascularization and ectopic bone formation.

CS, a simple, cheap and classic bioactive silicate ceramic, has been incorporated into many novel composite scaffolds^23, 25, 31^. For instance, a CS/PDLGA scaffold not only improves the degradability and mechanical properties of CS, but also enhances the ability of PDLGA to induce osteogenic differentiation and angiogenesis^32^. However, the degradative environment of pure CS is alkaline and contains a high concentration of Si ions, which inhibit cell activity. So CS is often used as a dopant instead^17, 24^. But the concentration of CS incorporated into scaffolds varies widely among studies^24, 31, 33^. Hence, to add the capacity of angiogenesis and osteoinduction to β-TCP, it is necessary to first determine the appropriate ratio of CS to incorporate into the scaffolds. Our studies showed that β-TCP scaffolds containing 5% CS had better biocompatibility than that of other ratios, and cell viability was not significantly different between β-TCP and 5%CS/β-TCP scaffolds. Besides, HUVECs and hBMSCs spread well on the surface of 5%CS/β-TCP scaffolds. And 5%CS/β-TCP scaffolds had acceptably stable pH (Supplementary Fig. S4). ICP was carried out and results showed that release of Si ions was observed. Next, to simulate the effects of scaffolds on cells *in vitro* and to assess the ability of 5%CS/β-TCP scaffolds to stimulate angiogenesis and osteogenesis, different from previous studies used scaffold extracts^22, 32^, 5%CS/β-TCP scaffolds were soaked in transwell inserts to assess the real-time effects of ion release on cell function. The 5%CS/β-TCP scaffolds were found to stimulate osteogenesis in hBMSCs and angiogenesis in HUVECs *in vitro*. These might be related to the stable release of Si ions during scaffolds degradation, and the Si ion concentration was consistent with that of other studies reporting effective concentration ranges^7, 22^.

The formation of new blood vessels depends on the ordered interaction of endothelial cells (ECs) with perivascular cells and some other cells^13, 34^. HUVECs have been shown to pre-vascularize silk scaffolds, and hBMSCs have been demonstrated to differentiate into perivascular cells *in vivo* ^14, 16^. Hence, co-cultured HUVECs and hBMSCs were selected as seed cells to promote vascularization in this study^16, 35, 36^. An effective Si ion concentration can enhance angiogenesis in co-cultured cells *in vitro* through stimulating the VEGF/BMP-2 paracrine pathway in HUVECs and hBMSCs^17^. So we investigated the real-time stimulation of angiogenesis in co-cultured HUVECs and hBMSCs seeded on Matrigel, and stimulated by 5%CS/β-TCP scaffolds physically separated in a transwell. Results showed that scaffolds stimulated the co-cultured system to abundantly secrete VEGF and BMP-2 to enhance angiogenesis. There are some differences between indirect and direct contact scaffolds. For example, hydrophilic properties affect cell adhesion and some special structures in the surface of scaffolds affect functional expression in cells^37–39^. Therefore, we co-cultured HUVECs and hBMSCs directly on scaffolds to investigate spread morphology and cell interactions on 3D materials. We found that co-cultured cells not only spread well on the surface of 5%CS/β-TCP scaffolds but also formed microcapillary-like structures on the scaffolds in 10 days. This might be related to the fact that 5%CS/β-TCP scaffolds stimulated co-cultured hBMSCs to secrete VEGF, a cytokine that can stimulate HUVEC angiogenesis^14, 17^. Besides, we found that hBMSCs were not only involved in tube and microcapillary-like structure formation, but were also evenly distributed among HUVECs on Matrigel and 3D scaffolds. Moreover, *in vivo* pre-seeded hBMSCs participated in the on-going process of new vessel maturation. Collectively, these data implied that 5%CS/β-TCP stimulated co-cultured cell angiogenesis and hBMSCs might act as perivascular cells to stabilize new vascular networks.

Subcutaneous pockets in nude mice, which are classic intradermal models of heterotopic bone formation with low blood supply and lack of natural bone-forming stem cells and growth factors^40^. This type of model is advantageous as it is beneficial to study the effects of exogenous cells and scaffolds with fewer interfering factors. So we examined angiogenesis and osteoinductivity *in vivo* by implanting these scaffolds into subcutaneous pockets in nude mice. Micro-CT and histology investigations revealed that 5%CS/β-TCP scaffolds stimulated vessels formation in comparison to pure β-TCP, 5%CS/β-TCP scaffolds seeded with HUVECs and hBMSCs further significantly enhanced and accelerated early vascularization, and pre-seeded hBMSCs participated in the on-going process of new vessel maturation *in vivo*. Besides, it is interesting to note that 5%CS/β-TCP scaffolds stimulated co-cultured cells to boost secretion of PDGF-BB and CXCL12 into the surrounding environment to enhance the migration of BMSCs. Of these factors, PDGF-BB was previously reported to stimulate proliferation and migration of perivascular and mesenchymal stem cells, while CXCL12 were shown to promote vascularization as well as hematopoietic stem cell recruitment and stabilization^30, 41^. These indicated that the secretion of PDGF-BB and CXCL12 in the surrounding environment might recruit host cells and initiate the formation of new vessels along with pre-seeded HUVECs and hBMSCs when implanted into subcutaneous^16, 29, 30^. Hence, 5%CS/β-TCP scaffolds seeded with HUVECs and hBMSCs could significantly enhance and accelerate early vascularization. Osteogenesis during bone remodelling was coupled with angiogenesis^4, 28, 42^. Rapidly formed new vessels would supply renewable autologous cells such as bone-forming stem cells and others to colonize on scaffolds with time increasing, and supply oxygen, nutrients and growth factors to these cells^2^. These might explain the ectopic bone formation at 8 weeks in the macropores of 5%CS/β-TCP scaffolds seeded with co-cultured cells, with significant ectopic bone formation at 12 weeks. However, some ectopic bone formed only at 12 weeks in 5%CS/β-TCP scaffolds. In addition, no ectopic bone and even fewer cells and new tissues were observed in the macropores of β-TCP scaffolds.

In conclusion, the macropores in these scaffolds promoted pre-seeded cells infiltrating, colonizing and proliferating on the whole scaffold^14, 43^. Si ions released from 5%CS/β-TCP scaffolds stimulated co-cultured cells to secrete growth factors into the surrounding environment. These may construct a pro-angiogenic tissue engineering microenvironment *in vitro* when cultured for several days^7, 17^. Once implanted *in vivo*, secreted PDGF-BB and CXCL12 could recruit host cells and initiate the formation of new vessels along with pre-seeded co-cultured cells^30, 44, 45^, and Si ions stably released from scaffolds could continuously stimulate new vessel formation. In turn, rapidly formed new vessels supply oxygen and nutrients to enhance survival rate of exogenous cells, and supply renewable autologous cells such as bone-forming stem cells and others to colonize on scaffolds with time increasing^4^. Therefore, there are much more cells colonization and new tissues formation in the macropores of scaffolds. Eventually, Si ions stably released from scaffolds and factors such as BMP-2 induce cells to form new bone, thus improving osteointegration^32^.

Nevertheless, there are some limitations associated with this study, and further research is needed to address related issues. First, to further estimate the reparative effects of 5%CS/β-TCP scaffolds with co-cultured BMSCs and endothelial cells on bone defects and orthotopic bone formation, larger animal model of bone defects should be established. Second, the specific molecular mechanisms that drive the secretion of PDGF-BB and CXC12 and the recruitment of BMSCs remain to be elucidated. Moreover, the specific role that 5%CS/β-TCP scaffold-derived ions and related signalling pathways play in cross-talk between co-cultured HUVECs and hBMSCs should be further characterized.

## Conclusion

In this study, we 3D-printed porous scaffolds with different ratios of CS and β-TCP. Of these, scaffolds with 5% CS were biocompatible, and stimulated angiogenesis and osteogenesis in comparison to β-TCP *in vitro*. Besides, we found 5%CS/β-TCP scaffolds enhanced co-cultured HUVECs and hBMSCs angiogenesis on Matrigel, stimulated co-cultured cells to form microcapillary-like structures on scaffolds, and stimulated pre-seeded co-cultured cells to secrete PDGF-BB and CXCL12 to recruit BMSCs *in vitro*. Moreover, 5%CS/β-TCP scaffolds promoted vascularization and induced ectopic bone formation in comparison to β-TCP, and synergized with co-cultured cells to further accelerate early vessels and ectopic bone formation when implanted subcutaneously in nude mice. These results suggest that doping with 5% CS might potentially improve the clinical therapeutic effect of pure β-TCP in bone repair. Besides, pre-seeding 5%CS/β-TCP scaffolds with co-cultured HUVECs and hBMSCs, to engineer a pro-angiogenic microenvironment *in vitro*, provides a potential strategy for vascularizing bone tissue engineering.

## Methods

### Fabrication of porous scaffolds

CS and β-TCP with particle sizes less than 38 μm were purchased from Kunshan Chinese Technology New Materials Co., Ltd. (Shanghai, China). β-TCP powders doped with 0%, 5%, 10%, 20%, 30%, 40%, and 50% CS (wt.%) were prepared in batches of 5 g, and mixed at 1,500 rpm for 30 min using a hybrid defoaming mixer (ARE-250, Thinky, Japan) as described previously^7^. Injectable powders for printing were prepared by combining 5 g of mixed powder with 3 g of polyvinyl alcohol solution (6 wt.%). Square scaffolds (7 mm × 7 mm × 3.5 mm) with interconnected 500 μm square channels were designed in CAD, allowing for approximately 30% shrinkage^7, 39^. The scaffolds were 3D-printed (Dresden, Germany) as described previously, dried at room temperature for 24 h, and sintered at 1,100 °C for 2 h^6^. The scaffolds obtained (5 mm × 5 mm × 2.5 mm) were sterilized using ethylene oxide^17, 46^, and halves (5 mm × 2.5 mm × 2.5 mm) were used in transwell and animal experiments.

### Cell isolation and culture

HUVECs were isolated according to published methods and cultured in Endothelial Cell Medium (Sciencell, USA)^47^. hBMSCs were isolated from bone marrow aspirates according to previous methods, and were cultured in α-MEM (Hyclone, USA) containing 10% FBS (Gibco, USA), 100 U/ml penicillin, and 100 μg/ml streptomycin^48^ HUVECs at passage 3–5 and hBMSCs at passage 2–4 were used for all experiments. All cells were maintained at 37 °C in a humidified incubator with 5% CO_2_.

### Cell viability assay

HUVECs and hBMSCs were separately seeded on various composite scaffolds in 24-well plates at 2 × 10^4^ cells/well and 3 × 10^4^ cells/well, respectively. Cells were allowed to adhere for 2 h, and then covered with 1 mL medium. Cell proliferation was measured after 1, 3, and 5 days by Cell Counting Kit-8 (Dojindo, Japan), following the manufacturer’s instructions. Data were collected in triplicate from three independent samples. HUVECs and hBMSCs were also seeded in empty 24-well plates at 2 × 10^4^ cells/well, respectively. Scaffolds were added after cells were allowed to adhere to the plate for 2 h. Cells were stained after three days using a Live/Dead Viability/Cytotoxicity Kit (Life Technologies, USA), which stains dead and viable cells with propidium iodide and Calcein AM, respectively. Finally, samples were imaged using an inverted fluorescence microscope equipped with a CCD camera (Olympus, Japan).

### Scaffold characterization

Phase composition was investigated by X-ray diffraction (Bruker-axs, Germany). Degradation was evaluated by shaking at 37 °C for 12 weeks in Tris-HCl pH 7.40, which was replaced every 7 days. Specimens were collected at various time points, rinsed with phosphate-buffered saline, and incubated for 24 h at 37 °C in L-DMEM. Extracts were prepared according to International Organization for Standardization 10993–12. The concentration of Ca and Si in extracts was determined by inductively coupled plasma optical emission spectroscopy (Thermo, USA), while pH was measured using a UB-7 pH meter (Denver Instruments, USA). Finally, scaffolds were dried in an oven for 24 h, and percentage weight loss was calculated.

### Scanning electron microscopy (SEM)

HUVECs and hBMSCs were seeded at 4 × 10^4^ cells on β-TCP or 5%CS/β-TCP scaffolds. After 48 h, samples were washed twice with phosphate-buffered saline (PBS), fixed with 4% paraformaldehyde (PFA) for 15 min, dehydrated using a series of ethanol solutions, dried at 36 °C for 4 h, sputter-coated with gold, and imaged by scanning electron microscopy (Quanta 250, FEI, USA).

### Alkaline phosphatase (ALP) staining and activity

HBMSCs were seeded at 1 × 10^5^ cells/well in 24-transwell plates (Corning, USA) containing β-TCP or 5%CS/β-TCP scaffolds in transwell inserts. Then, cells were induced with osteoblast-inducing conditioned media containing 0.05 mM L-ascorbic acid, 10 mM β-glycerophosphate, and 100 nM dexamethasone (Sigma, USA). ALP staining and activity were assessed after induction for 7 and 14 days using reagents from Beyotime Institute of Biotechnology (China), following the manufacturer’s instructions.

### *In vitro* tube formation

According to previous studies, co-cultured cells were mixed at a ratio of 5:1 (HUVECs: hBMSCs) in the study^16, 35, 36^. Growth factor-reduced Matrigel (50 μL, BD, USA) was plated in 96-transwell plates with 8 μm filters (Corning, USA), and seeded with HUVECs (3.6 × 10^4^), with a mix of cells suspension consisting of 6 × 10^3^ cells/well hBMSCs labelled with CellTracker^TM^ CM-Dil (Invitrogen, USA) and 3 × 10^4^ cells/well HUVECs transfected with lentivirus to express green fluorescent protein (GFP-HUVECs), and with GFP-HUVECs (3.6 × 10^4^), respectively. hBMSCs were labelled with CM-Dil according to the manufacturer’s instructions, while the lentiviral vector for transducing GFP was constructed according to published methods^49^. To study real-time stimulation of angiogenesis, β-TCP or 5%CS/β-TCP scaffolds were added to transwell inserts. After incubation at 37 °C for 4 h, HUVECs groups were stained with Calcein AM Fluorescent Dye (BD, USA), then tubes were imaged on an inverted fluorescence microscope. The number of formed tubes was calculated using Image Pro Plus 6.0.

### Confocal laser scanning microscopy

To investigate spread morphology and interactions between cells co-cultured on a scaffold, a mix of 1.5 × 10^5^ HUVECs and 3 × 10^4^ hBMSCs were seeded on β-TCP and 5%CS/β-TCP scaffolds in 1:1 (ECM:DMEM) medium. After 3 days, specimens were fixed with 4% PFA, permeabilized in 0.5% Triton X-100, stained with FITC-phalloidin (Sigma, USA), counterstained with DAPI (Beyotime Institute of Biotechnology, China), and visualized using a confocal laser scanning microscope (Leica TCS SP8, Germany). In this manner, viable cells were stained green, and hBMSCs were distinguishable from HUVECs by pre-staining with red CellTracker^TM^ CM-Dil.

CD31 expression in co-cultured cells was investigated at 10 days. Briefly, specimens were fixed with 4% PFA, permeabilized with 0.5% Triton-X 100, blocked with 1% bovine serum albumin in phosphate-buffered saline, probed overnight at 4 °C in blocking buffer supplemented with 1:100 CD31 antibody conjugated to Alexa Fluor 488 (Novus, USA), counterstained with DAPI, and imaged by confocal laser scanning microscopy (CLSM). As before, hBMSCs were distinguishable from HUVECs by pre-staining with CellTracker^TM^ CM-Dil.

### *In vitro* cell migration

Briefly, β-TCP or 5%CS/β-TCP scaffolds were placed in 24-transwell plates with 8 μm filters, and seeded with 6 × 10^4^ HUVECs or with a mix of 5 × 10^4^ HUVECs and 1 × 10^4^ hBMSCs. Cells were allowed to adhere to scaffolds for 1 h, and then covered with 1 mL α-DMEM. For comparison, HUVECs or co-cultured cells were also plated in empty 24-transwell plates without scaffolds. hBMSCs (5 × 10^3^) suspended in 150 μL α-DMEM were added to transwell inserts. Two hours later, the inserts were placed in the respective wells. To examine the effect of PDGFR and CXCR inhibition on cell migration, 10 μM imatinib (a PDGFR inhibitor) or 10 μM plerixafor (a CXCR inhibitor) from Dalian Meilun Biology Technology Co., Ltd. (China) were added to media, respectively. After 24 h, cells were fixed, and the upper surface of each filter was cleared of cells using cotton swabs. Cells were stained with crystal violet (Solarbio, China), and five random fields in each well were photographed. Migrating cells were quantified using Image Pro Plus 6.0.

### ELISA

Secreted VEGF, BMP-2, PDGF-BB, and CXCL12 in related media were quantified using ELISA kits (R&D Systems, USA) according to the manufacturer’s instructions. Data were collected in triplicate from three independent samples of each treatment group.

### ***In vivo*** implantation

A mix of 1 × 10^6^ HUVECs and 2 × 10^5^ hBMSCs was seeded on 5%CS/β-TCP scaffolds and cultured for five days *in vitro*. And then implanted into subcutaneous pockets in the dorsal region of 8 week-old male nude mice, which were anesthetized with 1.5% sodium pentobarbital during procedures. β-TCP scaffolds and 5%CS/β-TCP scaffolds were also implanted into subcutaneous pockets at the same time. Two dorsal subcutaneous pockets were created in each mouse. A total of 27 nude mice were used in this study, and were divided into three groups to be sampled at different time points, for a total of six samples of each material at each time point. Animals were sacrificed 4, 8, and 12 weeks after implantation. All protocols were approved by the Animal Care and Experiment Committee of Ninth People’s Hospital, Shanghai Jiao Tong University School of Medicine. And all methods were performed in accordance with relevant guidelines and regulations.

### Angiography

Mice were anesthetized with 1.5% sodium pentobarbital 4 and 8 weeks after scaffolds were subcutaneously implanted. The thoracic cavity was opened, a 22-gauge needle was inserted into the left ventricle, and 10 mL Microfil (Flow Tech, USA) per mice was perfused at 2 mL/min following perfusion with saline, as previously described^14, 50^. Mice were then kept at 4 °C for 24 h to coagulate the Microfil. Finally, implants were retrieved, fixed in 4% PFA, decalcified with 10% EDTA, imaged with μCT80 (Scano Medical, Switzerland), and reconstructed in 3D to visualize new blood vessels and tissues.

### Histology

Implants were retrieved at 8 and 12 weeks, fixed in 4% PFA, decalcified with 10% EDTA, embedded in paraffin, sectioned at 5 μm, and stained with haematoxylin-eosin and Masson’s trichrome to examine new vessels and new bone. To identify the origin of new vessels, immunohistochemical slices were stained with antibodies against human CD31 (Abcam 32457, UK) and human SMC (Abcam 10412, UK).

### Statistical analysis

Numerical data are reported as mean ± standard deviation, and were analyzed in SPSS 11.0 (SPSS, USA) by one-way analysis of variance followed by post-hoc Dunnett’s test, or by Student’s t test. *p* < 0.05 (*) and *p* < 0.01 (**) were considered to indicate statistical significance.

## Electronic supplementary material

Supplementary Information
