# Supplementary material for: 3D printed scaffolds of calcium silicate-doped β-TCP synergize with co-cultured endothelial and stromal cells to promote vascularization and bone formation
Source: Sci Rep. 2017 Jul 17;7:5588. doi: 10.1038/s41598-017-05196-1 (PMC5514115; doi:10.1038/s41598-017-05196-1)
Supplement: Supplementary file 1 — Supplementary Information [file 41598_2017_5196_MOESM1_ESM.doc]

Supplementary Information

**3D printed scaffolds of calcium silicate-doped β-TCP synergize with co-cultured endothelial and stromal cells to promote vascularization and bone formation**

Yuan Deng ^a, b, 1^, Chuan Jiang ^c, 1^, Cuidi Li ^a, b^, Tao Li ^b^, Mingzheng Peng ^b^, Jinwu Wang ^a, b, *^, Kerong Dai ^a, b, *^


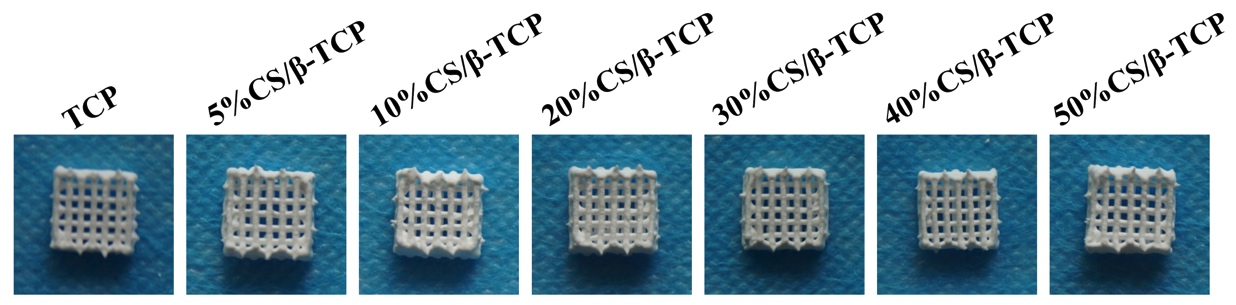


Fig. S1 Macroscopic of porous CS/β-TCP scaffolds contain different ratios of CS


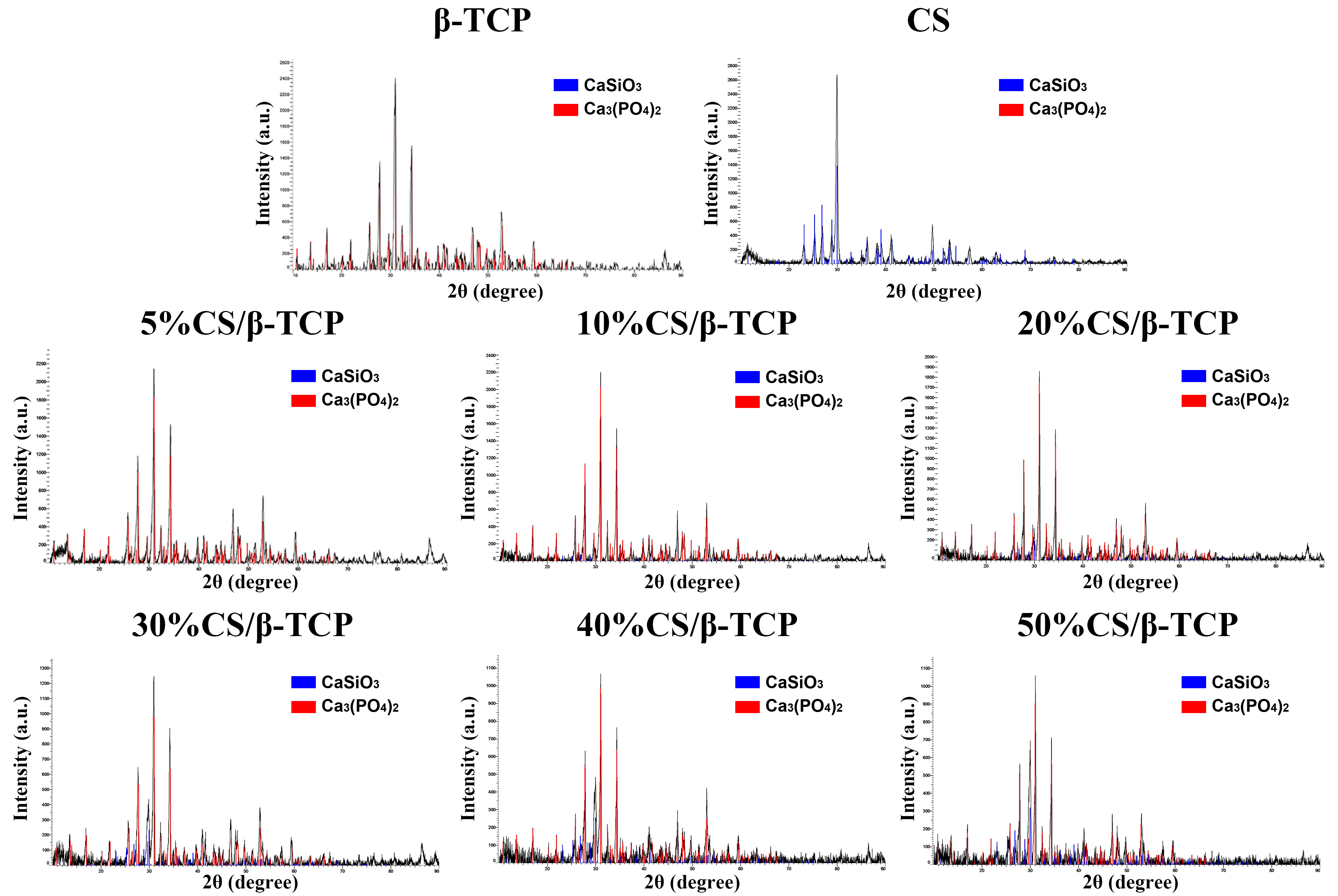


Fig. S2 X-ray diffraction patterns of porous composite scaffolds, including β-TCP, 5%CS/β-TCP, 10%CS/β-TCP, 20%CS/β-TCP, 30%CS/β-TCP, 40%CS/β-TCP, 50%CS/β-TCP.


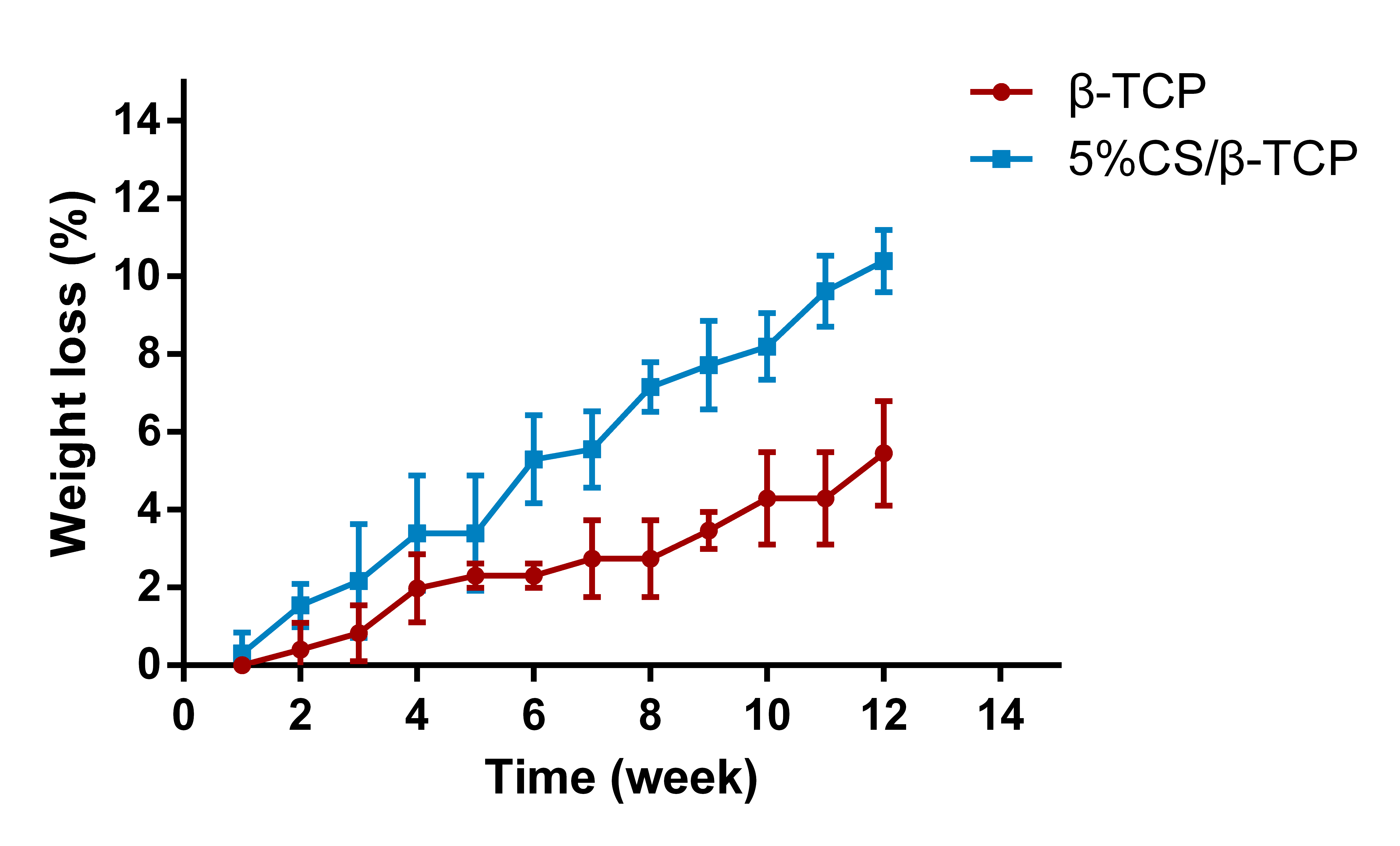


Fig. S3 Degradation profile of 5%CS/β-TCP scaffolds.


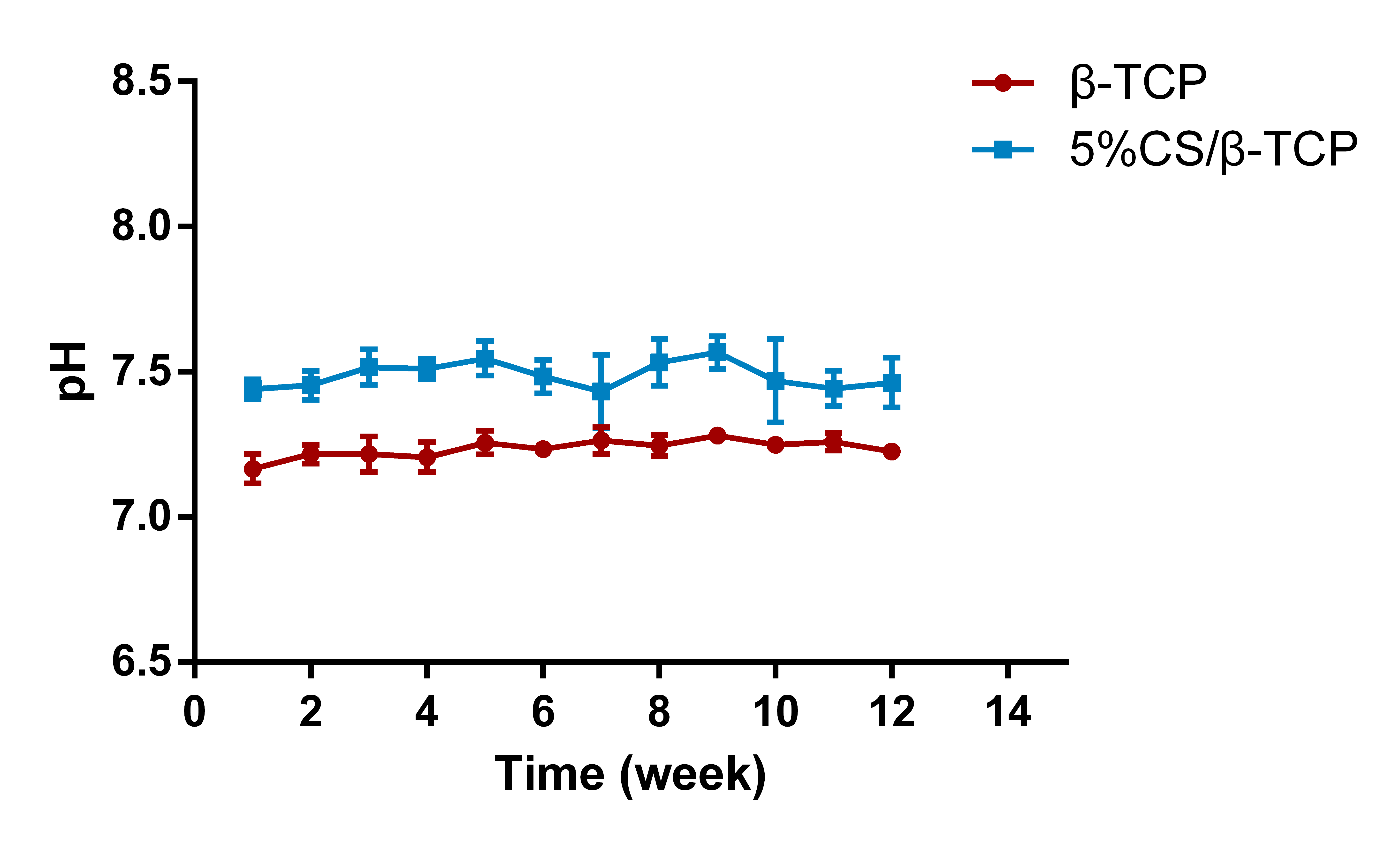


Fig. S4 PH of 5%CS/β-TCP scaffolds.
